# A Novel Cu_2_O/ZnO@PET Composite Membrane for the Photocatalytic Degradation of Carbendazim

**DOI:** 10.3390/nano12101724

**Published:** 2022-05-18

**Authors:** Liliya Sh. Altynbaeva, Murat Barsbay, Nurgulim A. Aimanova, Zhanar Ye. Jakupova, Dinara T. Nurpeisova, Maxim V. Zdorovets, Anastassiya A. Mashentseva

**Affiliations:** 1 The Institute of Nuclear Physics of the Republic of Kazakhstan, Almaty 050032, Kazakhstan; lilija310378@gmail.com (L.S.A.); nurgulim.a.a@gmail.com (N.A.A.); mzdorovets@inp.kz (M.V.Z.); 2 Department of Chemistry, L.N. Gumilyov Eurasian National University, Astana 010008, Kazakhstan; djakupova_zh@enu.kz (Z.Y.J.); nurpeisova_dt_1@enu.kz (D.T.N.); 3 Department of Chemistry, Hacettepe University, 06800 Ankara, Turkey; mbarsbay@hacettepe.edu.tr; 4 Department of Intelligent Information Technologies, The Ural Federal University, 620002 Yekaterinburg, Russia; 5 Engineering Profile Laboratory, L.N. Gumilyov Eurasian National University, Astana 010008, Kazakhstan

**Keywords:** copper(I) oxide, zinc oxide nanostructures, composite track-etched membranes, photocatalytic degradation of pesticide, carbendazim removal, wastewater pollutant, electroless template deposition

## Abstract

The extremely high levels of water pollution caused by various industrial activities represent one of the most important environmental problems. Efficient techniques and advanced materials have been extensively developed for the removal of highly toxic organic pollutants, including pesticides. This study investigated the photocatalytic degradation of the fungicide carbendazim (Czm) using composite track-etched membranes (TeMs) in an aqueous solution. Copper(I) oxide (Cu_2_O) and zinc oxide (ZnO) microtubes (MTs) were prepared using an electroless template deposition technique in porous poly(ethylene terephthalate) (PET) TeMs with nanochannels with a density of 4 × 10^7^ pores/cm^−2^ and diameter of 385 ± 9 nm to yield Cu_2_O@PET and ZnO@PET composite membranes, respectively. A mixed Cu_2_O/ZnO@PET composite was prepared via a two-step deposition process, containing ZnO (87%) and CuZ (13%) as crystalline phases. The structure and composition of all composite membranes were elucidated using scanning electron microscopy (SEM), atomic force microscopy (AFM), energy-dispersive X-ray spectroscopy (EDS), X-ray photoelectron spectroscopy (XPS) and X-ray diffraction (XRD) techniques. Under UV–visible light irradiation, the Cu_2_O/ZnO@PET composite displayed enhanced photocatalytic activity, reaching 98% Czm degradation, higher than Cu_2_O@PET and ZnO@PET composites. The maximum Czm degradation efficiency from aqueous solution was obtained at an optimal pH of 6 and contact time of 140 min. The effects of various parameters such as temperature, catalyst dosage and sample exposure time on the photocatalytic degradation process were studied. The degradation reaction of Czm was found to follow the Langmuir–Hinshelwood mechanism and a pseudo-first order kinetic model. The degradation kinetics of Czm accelerated with increasing temperature, and the activation energy (*E*_a_) levels were calculated as 11.9 kJ/mol, 14.22 kJ/mol and 15.82 kJ/mol for Cu_2_O/ZnO@PET, ZnO@PET and Cu_2_O@PET composite membranes, respectively. The reusability of the Cu_2_O/ZnO@PET catalyst was also investigated at different temperatures for 10 consecutive runs, without any activation or regeneration processes. The Cu_2_O/ZnO@PET composite exhibited degradation efficiency levels of over 50% at 14 °C and over 30% at 52 °C after 5 consecutive uses.

## 1. Introduction

Carbendazim (Czm) or methyl-2-benzimidazole carbamate is the most widely used active ingredient in the benzimidazole carbamate class of fungicides [1]. This fungicide has protective and curative activity against a wide range of fungal diseases and is the main degradation product of other benzimidazole fungicides, such as benomyl and thiophanate-methyl [2,3]. Czm is very stable in water (half-life of 5–26 days), wastewater, soil, crops and food, and is toxic to humans, animals and plants [4,5,6]. Since Czm is identified as a pollutant of water resources where it can accumulate, it is important to study the processes that lead to its detection [7,8], degradation [9] and removal. Currently, sorption [10], radiolytic [3,11] and catalytic degradation [12], oxidation and ozonation [13,14], membrane distillation [15], as well as a variety of microbiological methods [16] are successfully used to remove Czm from aqueous media.

The photocatalytic removal of various classes of organic and inorganic pollutants is one of the most widely used methods due to its high efficiency, low cost and simplicity. The current research in this area is aimed at developing new technologies for producing high-performance and low-cost catalysts. Various types of composite materials have attracted much research attention in the field of photocatalysis owing to their advantages, such as their design flexibility, improved physical and chemical properties and stability [17,18,19,20,21].

Various nanomaterials such as titanium dioxide [12,22], Fe/TiO_2_ [23] and Bi_2_S_3_/BiFeO_3_ [24] have been successfully used for photocatalytic degradation of Czm under UV irradiation. The high efficiency of Czm removal under UV irradiation makes this method one of the most promising, but increasing the diversity and efficacy of photoactive catalysts applicable to this reaction is a current relevant challenge. ZnO-based nanomaterials are among the most widely used in photocatalysis due to their high thermal conductivity, high exciton binding energy (60 MeV), high electron mobility and wide bandgap (3.2–3.4 eV) of zinc oxide [25,26]. A number of previous studies have shown that doping ZnO nanostructures with nanoscale forms of copper and CuO can significantly increase the catalytic activity in a variety of organic dye removal reactions [27,28,29,30,31].

Composite TeMs are a class of versatile micro- and nanoporous materials that contain ordered arrays of nano- or microtubes and are produced via electrochemical or electroless template deposition approaches [32,33]. The precise accuracy, pore sizes and high chemical stability of the polymer templates based on track membranes allow various types of simple and multicomponent compounds to be readily deposited in their pores. In our previous studies, composite TeMs based on metals of the copper subgroup demonstrated high catalytic activity in various reactions types in both batch and flow-through modes [34,35,36,37]. For example, Ag/PET composites have proven to be effective photocatalysts for the removal of methylene blue dye [38], while CuO-based MTs have been shown to exhibit high efficiency in the sorption of As(III) ions [34,39]. Some important advantages of such catalysts and sorbents are their ease of use, low cost and ease of production, as well as the possibility for multiple reuses without additional activation and regeneration procedures, as shown in previous studies [40,41].

The purpose of this work is to obtain composite track membranes based on chemically deposited copper(I) oxide (Cu_2_O@PET), zinc oxide (ZnO@PET) and a mixed composite (Cu_2_O/ZnO@PET) and to examine their catalytic activities in the degradation of pesticide Czm under UV light irradiation.

## 2. Materials and Methods

### 2.1. Chemicals

Copper sulfate pentahydrate (CuSO_4_·5H_2_O), zinc nitrate hexahydrate (Zn(NO_3_)_2_·6H_2_O), tin(II) chloride (SnCl_2_), palladium chloride (PdCl_2_), ethylenediaminetetraacetic acid (EDTA), dimethylamine borane (DMAB) and carbendazim (Czm) were all purchased from Sigma Aldrich (Schnelldorf, Germany and used without further purification. Deionized water (18.2 Mohm/cm, “Aquilon—D301” Aquilon, Podolsk, Russia) was used in all experiments.

### 2.2. Composite TeMs Synthesis

Electroless deposition of Cu_2_O and ZnO into nanochannels of PET TeMs (pore density: 4.2 × 10^7^ pores/cm^2^, pore diameter: 385 ± 9 nm) was carried out by applying the synthesis conditions presented in Table 1.

In the synthesis of single-component composites, the activated PET TeM sample was placed in a plating solution heated to the desired temperature, then after the deposition was complete the composite was washed in deionized water and dried in an oven at 60 °C for 20 min. To obtain a mixed composite (hereinafter referred to as Cu_2_O/ZnO@PET), the Cu_2_O/PET composite as the initial template was placed in a deposition solution containing Zn(NO_3_)_2_ and DMAB. Applying the conditions presented in Table 1 to prepare the ZnO@PET composite, ZnO was deposited on the precursor membrane to yield Cu_2_O/ZnO@PET. The amount of metallic catalyst deposit in the membrane template was determined using the gravimetric method and expressed in mg/cm^2^.

### 2.3. Characterization of the Structure and Composition of Composites

The pore size of the original template and the structural parameters of the MTs were determined by porometry method using the Hagen–Poiseuille Equation (1) [35]:

(1) $$Q=\frac{8\pi }{3MRT}\sqrt{\frac{nr^{3}\Delta p}{l}}$$

where Δ*p* is the pressure difference, MPa; *M* is the molecular mass of the gas, dyn × cm^–2^; *R* is the universal gas constant, erg/(mol × K); *n* is the number of microtubes per square centimeter of membrane area (template pore density); *l* is the membrane thickness, cm; and *T* is the temperature, K.

Morphological examinations and dimensional measurements of the resulting composites were performed using a JEOL JFC-7500F scanning electron microscope (SEM) ((Tokyo, Japan). Energy-dispersive X-ray spectroscopy (EDS) measurements were carried out using a Hitachi TM3030 (Hitachi Ltd., Chiyoda, Tokyo, Japan) microscope with a Bruker XFlash MIN SVE (Bruker, Karlsruhe, Germany) microanalysis system at an accelerating voltage of 15 kV.

The crystal structure of the nanoparticles was examined on a D8 Advance diffractometer (Bruker, Karlsruhe, Germany) in the angular range of 2θ 30–80° with a step of 2θ = 0.02° (measuring time: 1 s, tube mode: 40 kV, 40 mA). The mean size of crystallites was determined via the broadening of X-ray diffraction reflections using the Scherer formula [45]. The phase composition was determined using the Rietveld method, which is based on approximating the areas of the diffraction peaks and determining the convergence with reference values for each phase [46,47]. The volume fraction of the composite phase was determined using Equation (2) [48]:

(2) $$V_{\mathrm{admixture}}=\frac{RI_{\mathrm{phase}}}{I_{\mathrm{admixture}}+RI_{\mathrm{phase}}},$$

where *I*_phase_ is the average integral intensity of the main phase of the diffraction line, *I*_admixture_ is the average integral intensity of the additional phase, and *R* is the structural coefficient equal to 1.

XPS measurements were carried out using a Thermo Scientific K-Alpha spectrometer (Waltham, MA, USA) with a monochromatized Al Kα X-ray source (1486.6 eV photons) at a constant dwell time of 100 ms, pass energy of 30 eV with a step of 0.1 eV for core-level spectra and 200 eV with a step of 1.0 eV for survey spectra. The pressure in the analysis chamber was maintained at 2·10^−9^ Torr or lower. The binding energy (BE) values were referred to the C1s peak at 284.7 eV. Processing of the data was carried out using Avantage software (version 5.41, 2019, Waltham, MA, USA)

The surface morphology of the composite membranes was studied using a scanning probe microscope (SmartSPM-1000, NT-MDT, Novato, CA, USA) in semi-contact mode using a an NSG10 (TipsNano, Tallinn, Estonia) rectangular-shaped silicon cantilever (length 95 ± 5 μm, width 30 ± 5 μm, thickness 1.5–2.5 μm, probe tip radius = 10 nm, resonance frequency = 200 kHz). Initial scanning of the 10 × 10 μm^2^ sample area was performed at a speed of 5.0 μm/s. The average roughness was calculated from a scanning area of 3 × 3 μm^2^. The obtained data were processed and analyzed using IAPro software (version 3.2.2., 2012, NT-MDT, Novato, CA, USA).

### 2.4. Photocatalytic Degradation of Czm

To study the photocatalytic degradation reaction of Czm, 2 × 2 cm^2^ composite TeMs were placed in 50 mL of pesticide solution of a certain concentration, then stirred intensively in the dark for 60 min to achieve “catalyst–pesticide” adsorption equilibrium. The distance from the light source (Ultra-Vitalux 300W, Osram, Augsburg, Germany) to the surface of the solution was 15 cm. An aliquot of the reaction mixture with a volume of 1.5 mL was taken every 10–15 min and measured on a Specord-250 spectrophotometer (Jena Analytic, Jena, Germany) in the wavelength range of 200–400 nm. The degree of degradation (D%) was determined by Formula (3) [22]:

(3) $$D\%=\frac{C_{0}-C_{t}}{C_{0}}\times 100\%=\frac{A_{0}-A_{t}}{A_{0}}\times 100\%$$

where *C*_0_ and *C_t_* are Czm concentrations, and *A*_0_ and *A_t_* are absorbances at 285 nm at the beginning and time *t*, respectively. The effect of pH on the degradation efficiency of Czm was evaluated over the range of 4–9 at 30 °C, while other conditions were similar to those described above (the pH was adjusted with 0.1 M NaOH or 0.1 M HCl).

In order to examine the effect of the catalyst amount on the degradation efficiency of Czm, the composite area was varied from 4.0 to 10.0 cm^2^, while the Czm concentration was kept constant at 1.0 mg/L and the exposure time of the mixture was 160 min in all experiments.

The effect of temperature on the degradation efficiency of Czm was investigated in the temperature range of 14–52 °C (pH: 6, Czm concentration: 1.0 mg/L, catalyst size: 4.0 cm^2^).

## 3. Results

### 3.1. Composite Characterization

The techniques and methods of classical chemical copper plating processes are widely used to obtain different types of supported copper-based nanomaterials and copper coatings in modern materials science [49,50]. The diversity of reducing agents used in the copper deposition process [44,51,52,53] makes it possible to successfully vary both the morphology of the deposited copper structures and their composition and crystal structure [54,55]. At room temperature, reduction of copper(II) ions is possible only when formaldehyde is used as a reducing agent; deposition solutions based on formaldehyde have excellent stability and provide a high copper deposition rate. On the other hand, the high toxicity and volatility of formaldehyde and its identification by the World Health Organization as a substance with high carcinogenic and teratogenic activity raise serious concerns. The application of hypophosphite or hydrazine appears to be possible only at elevated temperatures, which limits their use in practice. In addition, boron-containing compounds, in particular dimethylamine borane (DMAB), are frequently used in chemical deposition processes [44,56]. In addition to the three covalent bonds with hydrogen, DMAB contains boron combined with trivalent nitrogen through the unshared electron pair of nitrogen by the donor-acceptor bonding mechanism. The main advantage of DMAB is the high stability of the molecule compared to sodium borohydride, which allows metal nanostructures to be chemically deposited under milder conditions—at a lower temperature (50–70 °C) and over a wide pH range (5–10).

**Figure 1:**
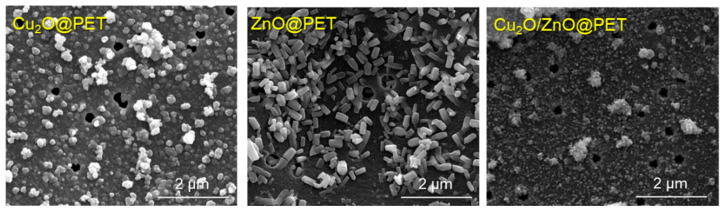
SEM images of the surfaces of synthesized composites.

Figure 1 shows electron micrographs of the synthesized composite TeMs. In these SEM images, besides the nanochannels that are still visible in places, the accumulation of nanoparticles is clearly visible as an abundant phase covering the entire surface of PET TeMs and the interior of the nanochannels. Based on the gravimetric analysis, the active phase loading rates per 1 cm^2^ of the composite were 0.28 mg, 0.13 mg and 0.35 mg for ZnO@PET, Cu_2_O@PET and Cu_2_O/ZnO@PET, respectively.

**Figure 2:**
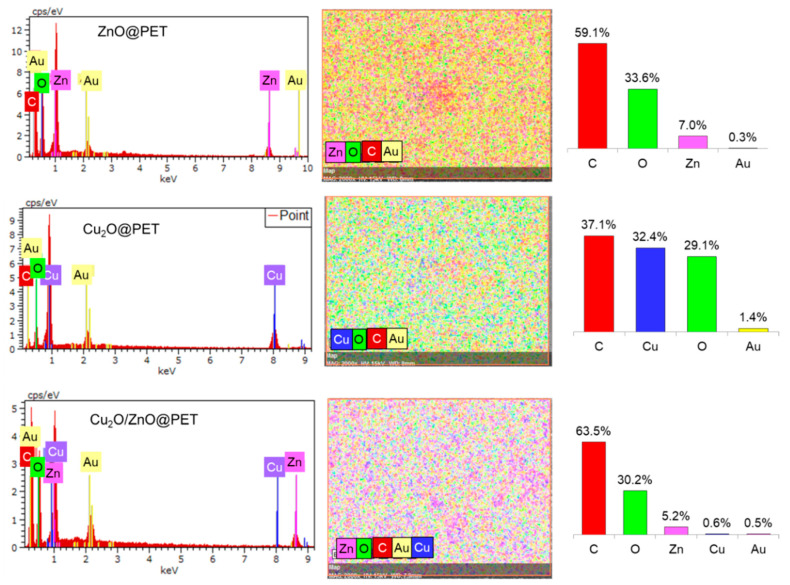
EDS spectra, elemental mappings and corresponding atomic percentages of the synthesized composite TeMs.

The elemental composition was investigated via energy-dispersive X-ray analysis (EDS), as seen in Figure 2. In the composition of all samples, intense carbon and oxygen peaks of the polymeric PET matrix and related elements (Cu and Zn) of the active phases (Cu_2_O and ZnO) were detected, as well as gold coated on the surface during the sample preparation stage. Besides the spectra, EDS mappings of the synthesized samples also showed the presence and uniform distribution of all detectable elements on the membrane surface. The trace amounts (0.3–1.4%) of gold found on the membrane surface can be attributed to the sample preparation for the SEM observation using the magnetron sputtering method.

**Figure 3:**
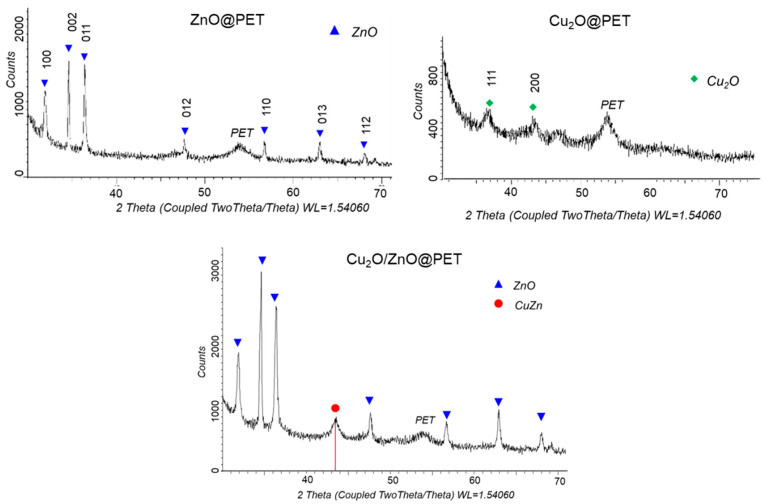
X-ray diffraction patterns of ZnO@PET, Cu_2_O@PET and Cu_2_O/ZnO@PET composites.

XRD was used to study the crystal structure of the obtained composite membranes (Figure 3). The XRD pattern of Cu_2_O@PET identified two diffraction peaks at 2θ equal to 36.46° and 43.85°, characteristic of the monoclinic structure of copper(I) oxide and attributed to planes (111) and (200), respectively (JCPDS: 01-073-6023, tenorite). The XRD diffractogram of ZnO@PET composite shows the characteristic diffraction peaks of ZnO phases at 2θ = 31.88° (100), 34.66° (002), 36.44° (101), 47.71° (102), 56.75° (110), 63.0° (103) and 68.18° (212) [57,58,59]. The identified planes are consistent with the JCPDS card of ZnO (JCPDS: 01-082-9744), indicating a hexagonal zincite structure (symmetry group P62mc (186)). In the XRD pattern of the mixed Cu_2_O/ZnO@PET composite, the 86.8% zinc oxide phase was identified.

Detailed data on the crystal structure of the synthesized composite TeMs are given in Table 2. According to the data obtained, while the average size of Cu_2_O crystallites in Cu_2_O@PET calculated by Scherer’s equation is 13 ± 4.5 nm, it is 44.2 ± 9.0 nm for ZnO crystallites in ZnO@PET membrane. For the mixed Cu_2_O/ZnO@PET composite, the crystallite size of the ZnO phase decreased by about 20% compared to the mono-component sample and was calculated as 31.5 ± 3 nm.

**Figure 4:**
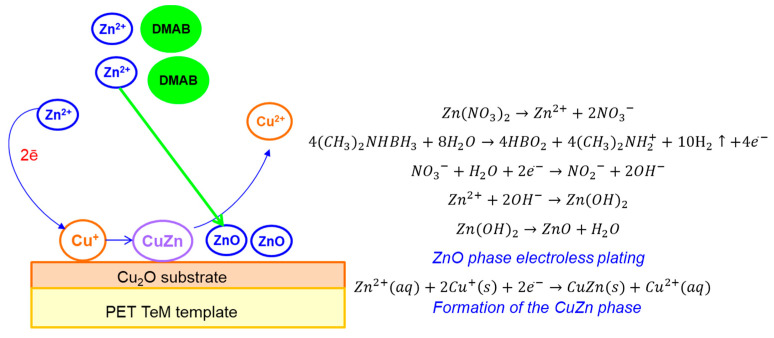
A schematic illustration of the synthesis of the Cu_2_O/ZnO@PET composite membrane based on the galvanic replacement reaction.

In addition to the main ZnO phase in the XRD pattern of the mixed Cu_2_O/ZnO@PET composite, an additional characteristic peak was observed at 2θ = 43.3°, attributed to the (110) plane of CuZn substitutional solid solution (SSS) phase (JCPDS: 01-071-5032 intermetallide zhanhengite). The crystallite size of this SSS phase was calculated as 11.97 nm based on the (110) peak. Figure 4 illustrates the proposed formation mechanism of CuZn SSS based on the galvanic replacement reaction on the Cu_2_O substrate under the condition of electroless plating at 70 °C. We consider that some Cu atoms of the minor component (Cu_2_O) are substituted for the atoms of the major ZnO component on the lattice positions normally occupied by oxygen atoms [60]. Along with CuZn phase formation, relevant reactions for ZnO plating in the presence of DMAB are presented in Figure 4.

**Figure 5:**
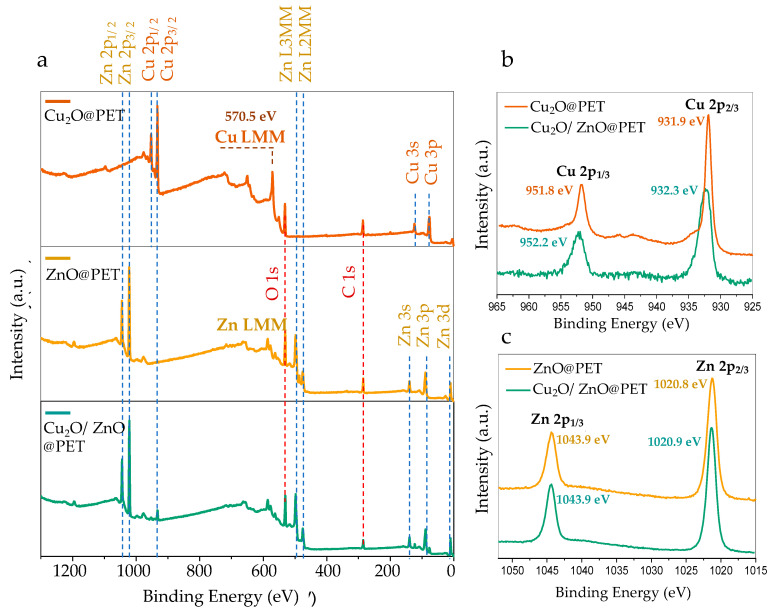
(**a**) Wide-scan XPS spectra of Cu_2_O@PET, ZnO@PET and Cu_2_O/ZnO@PET membranes. (**b**) Comparison of core-level Cu 2p XPS spectra of Cu_2_O@PET and Cu_2_O/ZnO@PET. (**c**) Comparison of core-level Zn 2p XPS spectra of ZnO@PET and Cu_2_O/ZnO@PET.

Oxides of copper (i.e., CuO and Cu_2_O) are promising materials because of the potential they offer for applications in catalysts, electronic interconnects, sensors and the corrosion of alloys, among others [34,61,62]. Determining the true oxidation state of copper in these systems is crucial to understand the chemical and physical properties, along with the practical behavior in terms of efficiency. X-ray photoelectron spectroscopy (XPS) is one of the most powerful techniques for determining the oxidation states of metals. Figure 5a shows the typical XPS wide-survey spectra of Cu_2_O@PET, ZnO@PET and Cu_2_O/ZnO@PET membranes. C and O peaks were detected around 285 eV and 531 eV, respectively, as shown in wide-survey spectra. The detected carbon is related to the carbon atom of the PET TeMs substrate. Aside from these two elements, all characteristic peaks of metal atoms (Cu and Zn) found in structures were identified in the spectra (Figure 5a).

It is generally accepted that XPS can be used effectively to distinguish between zero-valent metallic Cu, CuO and Cu_2_O. The metallic Cu and Cu_2_O are best distinguished from the X-ray-excited Cu LMM Auger peaks [63]. While the main LMM peak for metallic Cu is around 568 eV, in the case of Cu_2_O this peak appears at a significantly higher binding energy of around 570 eV [64]. As can be seen in Figure 5a, the LMM peak of the Cu_2_O@PET sample was observed at 570.5 eV, which clearly eliminates the metallic Cu state in the structure.

Copper(II) oxide (CuO) is characterized by high-intensity shake-up satellites with binding energies ~9 eV higher than the main Cu 2p_1/2_ and 2p_3/2_ peaks [63,64]. Additionally, in the case of CuO, the Cu 2p_1/2_ and 2p_3/2_ peaks are significantly wider, which is attributed to the shake-up process [63]. Figure 5b shows Cu 2p core-level spectra obtained from the as-prepared Cu_2_O@PET film and Cu_2_O/ZnO@PET membrane after the deposition process of ZnO. As seen in Figure 5b, only two Cu 2p core-level peaks were observed at 931.8 and 951.6 eV, which were attributed to Cu 2p_1/2_ and 2p_3/2_, respectively, in both spectra. The absence of well-detectable shake-up satellites in the Cu 2p core-level spectra excludes the presence of significant quantities of CuO and indicates that copper(I) oxide (Cu_2_O) is the predominant species. The binding energies of Cu 2p components of Cu_2_O@PET film (orange line, Figure 5b) are slightly lower than those of the mixed Cu_2_O/ZnO@PET membrane. This binding energy difference can be attributed to the chemical environment alterations of the surface copper atoms (to a depth of about 10 nm) following the electroless deposition of ZnO. Inspired by the results revealed by XRD, we attribute this change to the formation of the zhanghengite structure (CuZn) at the interface as a result of the atomic replacements in the lattice positions.

ZnO in the form of nanoscale materials can be regarded as one of the most important semiconductor oxides at present [65] and presents unique piezoelectric, pyroelectric [66] and catalytic [67] properties. As can be seen in Figure 5c, the Zn 2p core-level scans of ZnO@PET and Cu_2_O/ZnO@PET membranes have two components located at approximately 1043.9 and 1020.8 eV, which are attributed to Zn 2p_1/2_ and Zn 2p_3/2_, respectively [9,10]. The fact that the Cu_2_O-ZnO composite membrane (Cu_2_O/ZnO@PET) obtained by treating Cu_2_O@PET with Zn-plating solution and galvanization has peaks at almost the same location and shape as ZnO@PET indicates that the oxidation states of Zn are similar in both samples. The chemical valence of Zn at the surface in these samples corresponds to the +2 oxidation state and is perfectly compatible with the core-level Zn 2p spectrum of ZnO [68,69]. This result also shows that the possible CuZn phase mentioned in the XRD results is significantly less than ZnO in the mixed membrane (Cu_2_O/ZnO@PET) structure.

### 3.2. Photocatalytic Degradation of Czm

**Figure 6:**
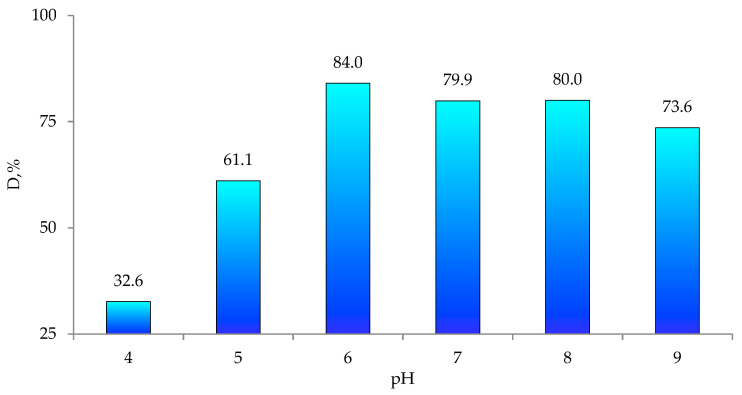
Variations in Cmz degradation degree (D, %) as a function of pH for ZnO@PET composite membrane.

The pH of the solution is one of the key parameters of photocatalytic processes. Changes in pH values affect the surface charge and degree of ionization of the catalyst, the electrostatic interactions between the catalyst surface and the reactant molecules and the distribution of functional groups in the catalyst’s active centers, as well as the chemical composition of the solution [70,71]. To find the efficiency of Czm degradation in the presence of ZnO@PET, we carried out a series of experiments in the pH range of 4–9, whereby the required pH level was adjusted using 1.0 M HCl_(aq)_ or NaOH_(aq)_. The pesticide concentration was 1.0 mg/L and the temperature was 30 °C. As can be seen from the data presented in Figure 6, the highest degradation efficiency of Czm was obtained at pH 6.0. When the pH was increased from 4 to 6, the removal efficiency increased from 32.52% to 83.7%. However, with a further increase in pH up to 9, the Czm removal efficiency remains practically unchanged. In addition, long-term operation of PET-based composites in alkaline environment leads to degradation of the polymer matrix. Therefore, pH 6.0 was chosen as optimal and further experiments were conducted at this pH.

**Figure 7:**
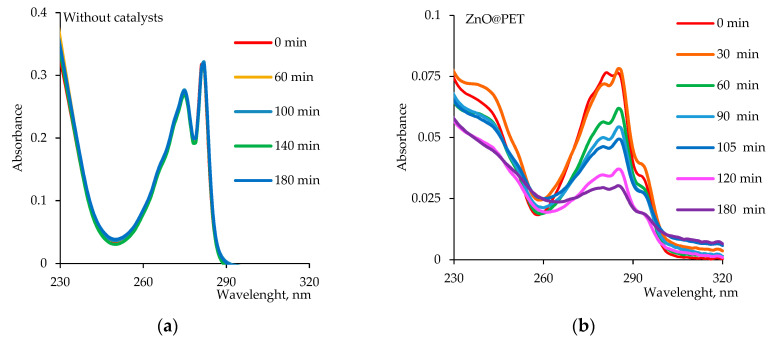
UV–vis absorption spectra of Czm (1.0 mg/L) as a function of time (**a**) without any catalyst and (**b**) in the presence of the 2 × 2 cm^2^ ZnO@PET composite membrane catalyst.

Figure 6 shows the time variation spectra of the absorbance of Cmz in the presence and absence of the ZnO@PET composite as a representative catalyst among 3 membranes. As can be seen in Figure 7a, in the absence of the catalyst, Cmz does not degrade over time, as can be seen from both the invariant forms of the absorption bands and their intensities. However, in the presence of ZnO@PET (Figure 7b), the decrease in intensity of the absorption bands and the significant changes in the band shapes indicate the degradation of Cmz and the formation of new degradation products.

**Figure 8:**
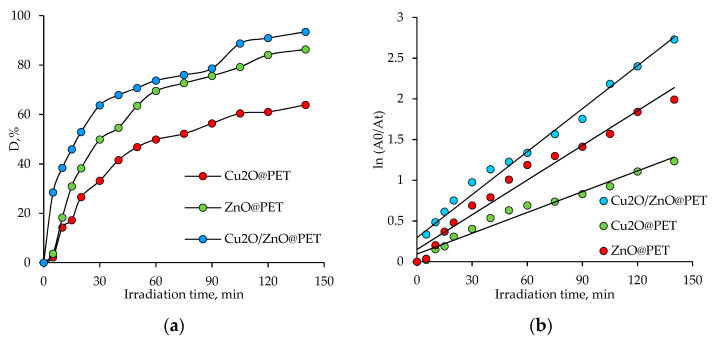
The variation in the degree of Czm degradation (D, %) as a function of irradiation time in the presence of different composite catalysts (2 × 2 cm^2^, Czm feed, concentration: 1.0 mg/L) (**a**), and Langmuir–Hinshelwood plots for photodegradation of Cmz catalyzed by different composites (**b**).

The time-dependent photodegradation efficiency of Czm in the presence of the same amount of composite catalysts (2 × 2 cm^2^), taking into account the maximum absorbance peak around 285 nm, is shown in Figure 8a. After 140 min of irradiation, 65.3% and 86.4% of the pesticide were degraded in the presence of Cu_2_O@PET and ZnO@PET catalysts, respectively, while more than 93% of Czm was decomposed when mixed Cu_2_O/ZnO@PET catalyst was used for the same reaction time. The catalytic photodegradation of Cmz was quantitatively estimated by comparing the apparent reaction rate constants ( $k_{a}$ ) calculated from the first-order rate equation derived from the Langmuir–Hinshelwood model, expressed as follows [12,14]:

(4) $$\ln\left(\frac{C_{0}}{C}\right)=k_{a}t$$

where *C*_0_ is the initial concentration of Czm (mg/L), *C* is the concentration of the Czm at time *t*, *t* is the irradiation time (min) and $k_{a}$ is the reaction rate constant (min^−1^).

The Langmuir–Hinshelwood plots (Figure 8b) obtained for the degradation of Cmz in the presence of different composite catalysts (2 × 2 cm^2^) were all linear, suggesting that the photodegradation of Cmz follows the pseudo-first order reaction kinetics. Accordingly, the calculated $k_{a}$ for the mixed Cu_2_O/ZnO@PET was 1.76 × 10^−2^ min^−1^, while lower values of 1.42 × 10^−2^ and 0.85 × 10^−2^ min^−1^ were found for the single-component ZnO and Cu_2_O composite catalysts, respectively. In line with these results, it can be said that the mixed composite membrane catalyzes the UV-mediated degradation of Cmz more efficiently and rapidly.

**Figure 9:**
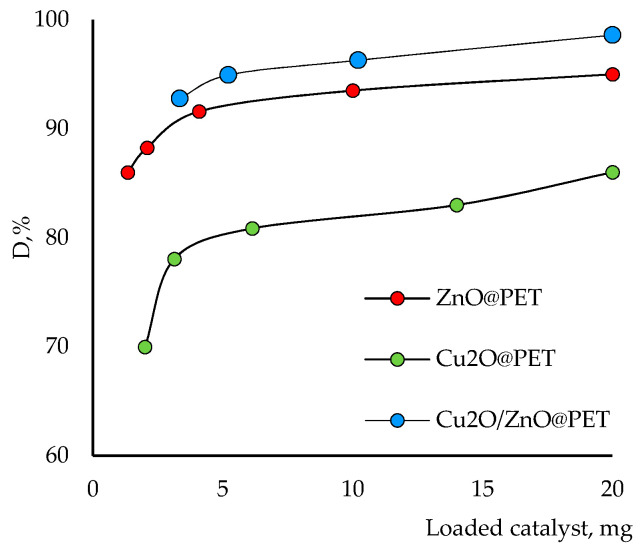
Effects of the catalyst amount on the degradation efficiency of Czm (1.0 mg/L, time 160 min and pH 6.0).

The catalyst dosage is also an important parameter for optimizing working conditions and comparing the effectiveness of catalysts. Therefore, the effects of the active phase of the composite catalyst dosage on the degradation of Czm were investigated at different dosages from 1.3 to 20.0 mg and the results are given in Figure 9. The results indicated that the amount of degradation gradually increased with increasing catalyst dosage over the studied range. On the one hand, as expected, 20 mg catalyst showed the highest photocatalytic degradation efficiency due to the increase in the number of active sites and the high surface area of the composite catalyst. On the other hand, consistent with previous observations, the mixed catalyst showed higher degradation efficiency than the others at all doses, followed by ZnO@PET and Cu_2_O@PET.

**Figure 10:**
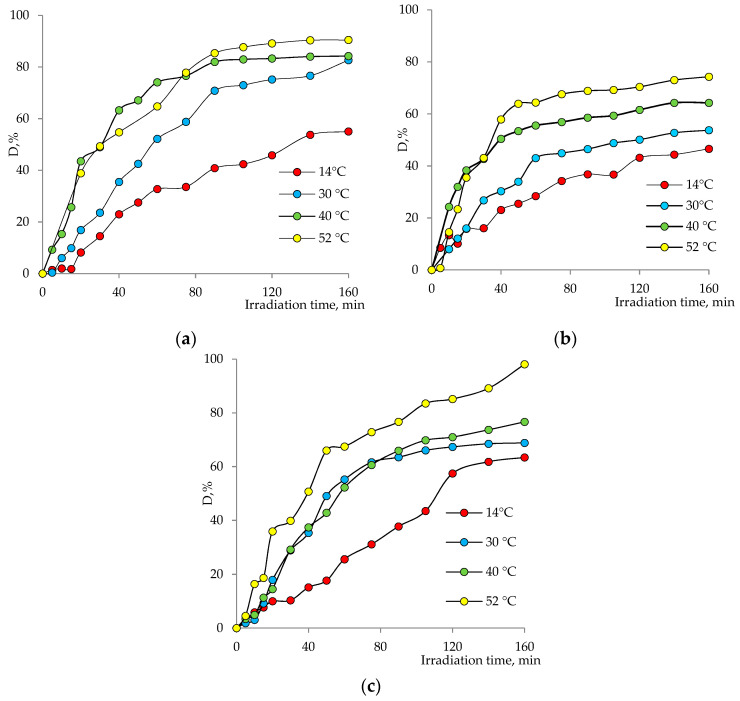
Variation in Czm degradation degree with irradiation time at different temperatures in the presence of (**a**) ZnO@PET, (**b**) Cu_2_O@PET and (**c**) Cu_2_O/ZnO@PET composite membranes.

The effect of temperature on the degradation efficiency of Czm was investigated in the temperature range of 14–52 °C (pH = 6, Czm concentration: 1.0 mg/L). The levels of dependence of the pesticide degradation degree on the irradiation time for different temperature regimes and catalysts are shown in Figure 10a–c. From the presented data, it can be said that for all catalyst systems, there is a general tendency to increase in the amount of degradation with an increase in temperature, and after a certain period of time, the degradation tends to reach almost equilibrium at all temperatures. The highest activity in the degradation of Czm over the entire temperature range was demonstrated by the mixed composition catalysts, which catalyzed the degradation of almost all of the contaminant in the medium after 160 min at 52°. Cu_2_O@PET and ZnO@PET, on the other hand, failed to degrade more than 74% and 90% of Cmz, respectively, under similar reaction conditions. In other conditions, it is also possible to say that the composite catalyst, in general, performs more effectively. Moreover, since all composite catalysts demonstrate undeniable catalytic activity even at very low temperatures (e.g., 14 °C), the developed membrane catalysts seem suitable to be used for wastewater treatment without preheating, especially considering their ease of use. While the lowest values of the reaction rate constant were calculated for Cu_2_O based composite, Cu_2_OZnO@PET gave the highest rate constants.

**Figure 11:**
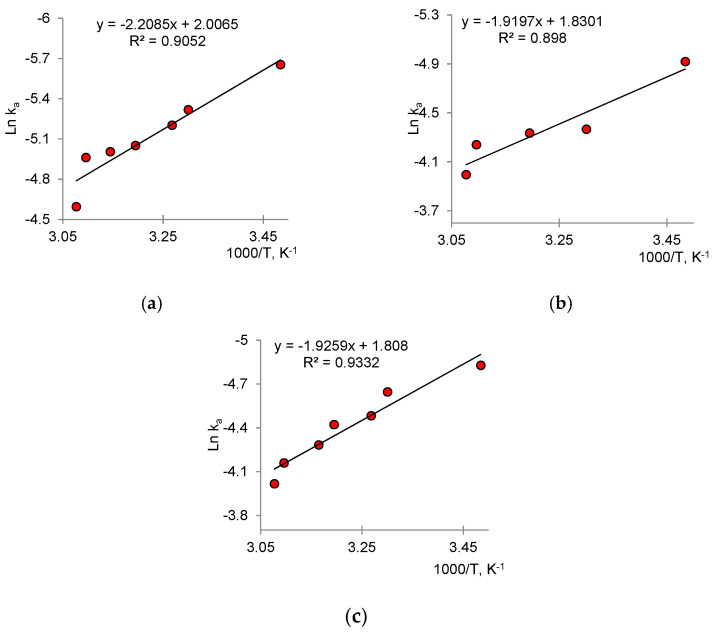
Arrhenius graphs for determining *E*_A_ for (**a**) Cu_2_O@PET, (**b**) ZnO@PET and (**c**) Cu_2_O/ZnO@PET composite membranes.

The activation energy *E*_A_ was calculated using the Arrhenius equation (Equation (5)) and determined graphically from the relation ln *k_a_* -(1000/T) (Figure 11):

(5) $$\ln k_{a}=\ln A-\frac{E_{A}}{RT},$$

where *k_a_* is the rate constant, min^−1^; *A* is the pre-exponential multiplier; *E*_A_ is the activation energy, J/mol; *R* is the gas constant, equal to 8.314 J/molK; *T* is the temperature, K.

The lowest *E*_A_ value was obtained for the mixed composites of Cu_2_O/ZnO@PET (please see Table 3). Unfortunately, it is not possible to compare the data obtained with previous studies, because similar thermodynamic parameters for the photocatalytic degradation of Czm have not been reported before. However, the low *E*_A_ values obtained for all three catalysts allow us to judge their high catalytic activities.

The Eyring equation [71,72] was used to calculate activation enthalpy (Δ*H*^≠^; kJ/mol) and entropy (Δ*S*^≠^; J mol/K) from the slope and intercept of the ln(*k*_app_/*T*) versus 1/*T* graph, respectively (6):

(6) $$\ln\left(\frac{k_{a}}{T}\right)=\ln\left(\frac{k_{B}}{h}\right)+\left(\frac{\Delta S^{\neq }}{R}\right)-\left(\frac{\Delta H^{\neq }}{RT}\right)$$

where $k_{B}$ and *h* are the Boltzmann and Planck constants, respectively. Given the positive Δ*H*^≠^ and negative Δ*S*^≠^ values in Table 3, it appears that endothermic interactions and a decrease in entropy occur at the solid–liquid interface during the degradation of Czm on the surfaces of the studied composite TeMs.

Table 4 compares our results with previously published data on the catalytic activity of various types of catalysts used for the degradation of Czm. It should be noted that it is rather difficult to directly compare the data from various studies, as some determining parameters such as the amount of loaded catalyst, the initial concentration of Czm in the tests and the type and size of catalyst are not exactly the same. Nevertheless, it can easily be said that our results compete closely with existing alternatives and that the obtained composite membranes are promising considering their practicality and high surface areas.

**Figure 12:**
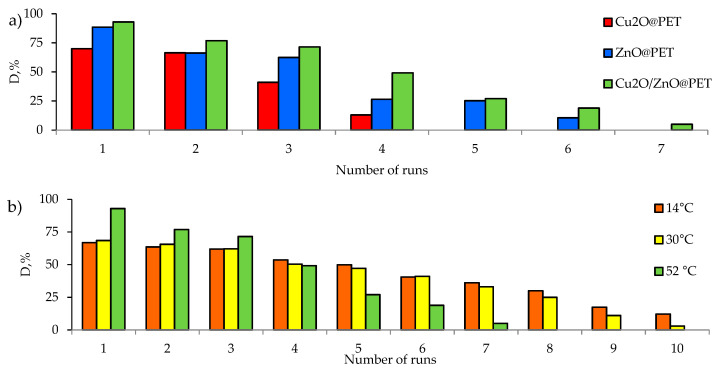
Variation in the degradation of Czm (1.0 mg/L) with different composite TeM catalysts for several consecutive runs at 52 °C (**a**), along with the reusability of Cu_2_O/ZnO@PET at different temperatures (**b**).

An important parameter for catalysts is the stability of their performance. Reusability is one of the most important requirements for catalysts. An important advantage of using supported catalysts is that unlike non-supported powders and nanoparticles (except magnetic ones), they can be easily removed from the reaction mixture. All synthesized composites were tested in several consecutive cycles. The results are shown in Figure 12a. The Cu_2_O-based catalyst completely lost its activity after 4 test cycles. The ZnO@PET composite catalyzed the degradation of approximately 88.5% of the pesticide after the 1st cycle, while it was able to remove only 25% and 10% in the 5th and 6th cycles, respectively. Cu_2_O/ZnO@PET catalyzed the degradation of 26% of Czm after the 6th cycle, while only 5% of Cmz was removed at cycle 7. These significant decreases observed especially after the 3rd cycle are attributed to the washout of the active phase from the polymer template due to intensive agitation of the reaction mixture; the weight reduction of Cu_2_O/ZnO@PET composite was only 7% after the 1st cycle, but the mass losses exceeded 19% and 45% after the 3rd and 7th cycles, respectively.

The changes in the crystal structures of the catalysts after the last test cycle were investigated by XRD. It was found that there were significant increases in crystallite size due to the leaching of smaller nanoparticles from the structures. For example, after the 4th test cycle, the degree of crystallinity for Cu_2_O@PET composite decreased from 53.4% to 42.1%, while the size of the Cu_2_O crystallites increased from 13 to 35 nm. In the case of Cu_2_O/ZnO@PET composites, it was determined that a completely amorphous structure remained after the 7th test cycle. However, when repeated cycles were performed at lower temperatures (Figure 12b), we found that the performance of the Cu_2_O/ZnO@PET composite catalyst remained relatively stable over longer test cycles. The degradation degree, which was actually higher at 52 °C in the first cycle, was outstripped by the lower temperature regimes at the end of the 4th cycle. We attribute this to less catalyst leakage at lower temperatures. As seen in Figure 12b, at lower temperatures, the catalyst was still active at the end of the 10th cycle. Therefore, it can be recommended to use composite catalysts in relatively low-temperature regimes such as 14 to 30 °C for longer repeated operations.

**Figure 13:**
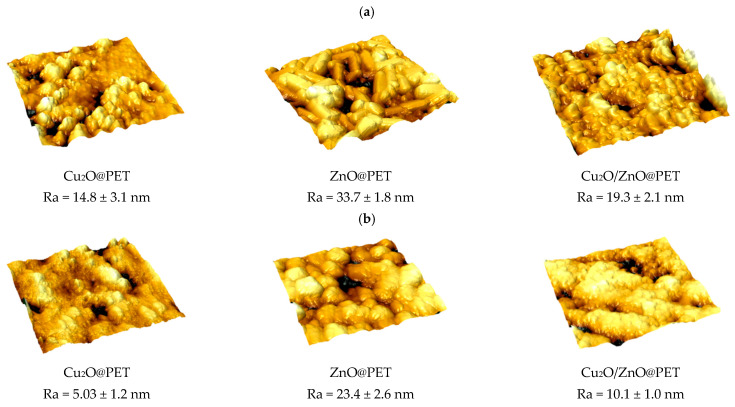
Atomic force microscopy (AFM) images of the surfaces of composite catalysts before (**a**) and after the 4th run of the catalyst treatment (**b**), with a scanning area of 3 × 3 µm^2^.

To further examine the changes in structure of composite membranes after catalytic studies, surface topography and roughness (R_a_) were investigated using atomic force microscopy (AFM) at a scan size of 3 × 3 μm. Figure 13 presents the 3-dimensional images acquired from the AFM analysis and the calculated R_a_ values for each image, before use in the photocatalytic degradation reaction and at the end of the 4th test cycle. Average roughness values were calculated for at least 10 images of 512 × 512 points taken from different locations. In the AFM images, the leakage of the active nanosized catalytic phase from the membrane surface is manifested by the decreasing amount of nanoparticles in the images. In addition, leakage directly affects the surface roughness and causes a significant reduction in the R_a_ value of each composite membrane at the end of its use in the 4th catalytic cycle, which explains the decreased performance obtained in repeated uses.

## 4. Conclusions

Herein, a novel mixed Cu_2_O/ZnO@PET composite was synthesized, along with porous composite PET TeMs based on copper(I) and zinc oxides, using the electroless template synthesis method. The structures of all samples were extensively characterized. The Cu_2_O/ZnO@PET composite was found to consist of a ZnO phase (86.8%) and a mixed phase of Cu_2_O, as well as a substitutional solid solution with a crystal structure of zhanhengite intermetallide (CuZn). The catalytic performances of the composites were studied on the photodegradation reaction of a toxic pesticide, carbendazim. The composite membrane catalysts exhibited higher degradation efficiency at pH 6.0. The mixed composite (Cu_2_O/ZnO@PET) catalyzed the degradation more effectively compared to Cu_2_O@PET and ZnO@PET, and decomposed more than 93% of Czm after 140 min of irradiation.

Photodegradation of Cmz was found to follow the pseudo-first-order reaction kinetics. The highest reaction rate constant (1.76 × 10^−2^ min^−1^) and the lowest activation energy (11.9 kJ/mol) were calculated for the mixed Cu_2_O/ZnO@PET composite. We speculate that the presence of both active phases (ZnO and Cu_2_O) and the intermetallide (CuZn) at their interfaces causes a synergistic catalytic effect in the photodegradation of Cmz in the case of mixed Cu_2_O/ZnO@PET composites. The stability of the catalyst properties at different temperatures was also investigated by performing repeated test cycles, and it was found that the activity was more effectively preserved at lower temperatures. A decrease in the efficiency of the reaction was observed after repeated test cycles, depending on several factors, such as washing out the catalytic phase with nanoscale active sites from the membrane surface, decreases in crystallinity and increases in crystallite size. In light of these results, the synthesized composite catalysts can be considered as effective alternatives in the photodegradation of Cmz, especially considering their ease of use.

## Figures and Tables

**Table 1:** Synthesis conditions of composite TeMs.

Composite	Template Preparation Conditions	Synthesis Conditions		Composition of the Deposition Solution	Ref.
		T, °C	Deposition Time, min		
ZnO@PET	Sensitization—SnCl_2_ (20 g/L), HCl (60 mL/L, 37%) 15 min, RT.	70	20	0.0013 M Zn(NO_3_)_2_, 0.05 M DMAB; pH = 6.0	[42,43]
Cu_2_O@PET	Activation—PdCl_2_ (0,1 g/L), HCl (20 mL/L, 37%), 15 min, RT	45	30	CuSO_4_ × 5H_2_O: 10 g/L; EDTA: 14 g/L; DMAB: 6 g/L; pH = 1.85	[44]
Cu_2_O/ZnO@PET	Cu_2_O@PET composite was used as a template	70	20	0.0013 M Zn(NO_3_)_2_, 0.05 M DMAB; pH = 6.0	-

**Table 2:** Changes in the crystal structures of the composites according to XRD data.

Composite	Phase Content	Type of Structure	Group of Symmetry	(hk) *^a^*	2θ°	d, Å *^b^*	L, nm *^c^*	Cell Parameter, Å ^*d*^	FWHM *^e^*	Crystall. Degree, %
Cu_2_O@PET	Cu_2_O/ 100%	Cubic	Fm-3m (225)	111	36.5	2.462	10.13	a = 4.224	0.917	53.4
				200	43.2	2.093	16.53		0.573	
ZnO@PET	ZnO/ 100%	Hexagonal	P62mc (186)	100	31.9	2.805	33.93	a = 3.240, c = 5.185	0.271	62.4
				002	34.7	2.586	53.08		0.174	
				011	36.4	2.464	43.39		0.214	
				012	47.7	1.905	56.90		0.170	
				110	56.7	1.621	48.70		0.206	
				013	63.0	1.474	37.96		0.273	
				112	68.2	1.374	35.32		0.302	
Cu_2_O/ZnO@PET	ZnO/ 86.8%	Hexagonal	P62mc (186)	100	31.9	2.805	28.71	a = 3.236, c = 5.177	0.320	77.5
				002	34.7	2.585	36.14		0.256	
				011	36.4	2.467	28.95		0.321	
				012	47.6	1.908	29.83		0.323	
				110	56.6	1.624	28.82		0.348	
				013	62.8	1.477	35.76		0.289	
				112	67.9	1.378	32.13		0.331	
	Cu_2_O and CuZn *^f^*/ 13.2%	Cubic	Pm-3m (221)	110	43.3	2.088	11.97	a = 2.940	0.794	

*^a^* Miller indices for corresponding planes; *^b^* spacing between planes; *^c^* average crystallite size; *^d^* crystal lattice parameter; *^e^* full-width at half-maximum; *^f^* this phase includes Cu_2_O and CuZn, although the tabulated data were calculated on the basis of the characteristic (110) peak of CuZn.

**Table 3:** Thermodynamic parameters of the degradation reaction of Czm.

Composite	*E*_A_, kJ/mol	ΔH, kJ/mol	ΔS, J/(mol × K)
ZnO@PET	14.22 ± 1.34	15.96 ± 1.50	−182.35 ± 9.76
Cu_2_O@PET	15.82 ± 1.67	18.36 ± 2.03	−180.88 ± 8.95
Cu_2_O/ZnO@PET	11.90 ± 1.03	16.01 ± 1.97	−182.53 ± 9.22

**Table 4:** Catalytic activity levels of various nanosized materials in the degradation of Czm.

Catalyst	Amount of Loaded Catalyst, g/L	Details of Catalytic Experiments	Catalysts Efficiency			Ref.
			D, %	k_a_, min^−1^	E_A_, kJ/mol	
Fe/TiO_2_ (2 wt%)	1.0	Sunlight, T = 25 °C, Czm = 8.0 mg/L	98.5	0.08	-	[73]
TiO_2_	1.0	UV, T = 20 °C, Czm = 40.0 mg/L	42.8	-	-	[74]
	0.07	UV, T = 25 °C, Czm = 10.0 mg/L, pH = 6.7	91.0	0.03	-	[12]
TiO_2_/UV/ozone	1.0	UV, O_3—_0.48 g/h, T = 20 °C, Czm = 40.0 mg/L	69.2	-	-	[74]
P25 TiO_2_	1.0	UV, Czm = 10 mg/L, pH = 6.5	85.0	0.065	-	[75]
Bi_2_S_3_/BiFeO_3_	0.5	UV, Czm = 10 mg/L	95.0	-	-	[24]
ZnO@PET	0.022	UV, T = 52 °C, pH = 6.0, Czm = 1.0 mg/L,	90.6	0.019	14.22 ± 1.34	This study
Cu_2_O@PET	0.011		74.3	0.010	15.82 ± 1.67	
Cu_2_O/ZnO@PET	0.032		98.1	0.021	11.90 ± 1.03	

## Data Availability

Not applicable.
