# Correction: Altynbaeva et al. A Novel Cu_2_O/ZnO@PET Composite Membrane for the Photocatalytic Degradation of Carbendazim. *Nanomaterials* 2022, *12*, 1724

**DOI:** 10.3390/nano15110808

**Published:** 2025-05-28

**Authors:** Liliya Sh. Altynbaeva, Murat Barsbay, Nurgulim A. Aimanova, Zhanar Ye. Jakupova, Dinara T. Nurpeisova, Maxim V. Zdorovets, Anastassiya A. Mashentseva

**Affiliations:** 1 The Institute of Nuclear Physics of the Republic of Kazakhstan, Almaty 050032, Kazakhstan; lilija310378@gmail.com (L.S.A.); nurgulim.a.a@gmail.com (N.A.A.); mzdorovets@inp.kz (M.V.Z.); 2 Department of Chemistry, L.N. Gumilyov Eurasian National University, Astana 010008, Kazakhstan; djakupova_zh@enu.kz (Z.Y.J.); nurpeisova_dt_1@enu.kz (D.T.N.); 3 Department of Chemistry, Hacettepe University, 06800 Ankara, Turkey; mbarsbay@hacettepe.edu.tr; 4 Department of Intelligent Information Technologies, The Ural Federal University, 620002 Yekaterinburg, Russia; 5 Engineering Profile Laboratory, L.N. Gumilyov Eurasian National University, Astana 010008, Kazakhstan

In the original publication [1], there was a mistake in Figure 13 as published. The atomic force microscopy (AFM) image of Cu_2_O/ZnO@PET before the catalysis process is duplicated with the AFM image of ZnO@PET after the catalysis process. The corrected Figure 13 appears below. 

The city listed for affiliations 2 and 5 in the original publication was incorrect and has been corrected to “Astana”.

The authors state that the scientific conclusions are unaffected. This correction was approved by the Academic Editor. The original publication has also been updated.

## Figures and Tables

**Figure 13:**
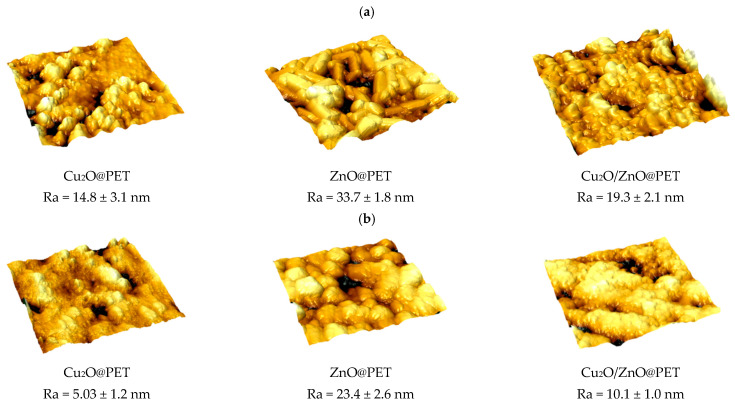
Atomic force microscopy (AFM) images of the surfaces of composite catalysts before (**a**) and after the 4th run of the catalyst treatment (**b**), with a scanning area of 3 × 3 µm^2^.
